# Safety and Effectiveness of an Investigational Insulin Delivery Device Providing Basal/Bolus Therapy with Rapid-Acting or Regular Human Insulin in Adults with Type 2 Diabetes

**DOI:** 10.1089/dia.2019.0356

**Published:** 2020-04-27

**Authors:** Ronnie Aronson, Edward Mahoney, Drilon Saliu, David Sze, Didier Morel, Leya Bergquist, Laurence Hirsch

**Affiliations:** ^1^ LMC Diabetes and Endocrinology, Toronto, Canada.; ^2^ Medical Affairs, Becton, Dickinson and Company, Franklin Lakes, New Jersey.; ^3^ Global Clinical Development, Becton, Dickinson and Company, Le Pont de Claix, France.; ^4^ Human Factors Engineering, R&D, Becton, Dickinson and Company, Franklin Lakes, New Jersey.

**Keywords:** Glycemic control, Insulin delivery device, Prospective study, Type 2 diabetes

## Abstract

***Background:*** This study undertook to assess usability, 24-h glycemic profiles, and safety of an investigational basal/bolus insulin delivery device (IDD) providing rapid-acting or regular human insulin (RHI) for people with type 2 diabetes (T2D) transitioning from multiple daily insulin injections (MDIs).

***Methods:*** This prospective, single-center, open-label two-period study enrolled adults with T2D and glycated hemoglobin (HbA1c) 7%–11% (53–97 mmol/M). Participants continued the usual MDI therapy during a 2- to 3-day in-clinic MDI period and then within 7 days were switched to the IDD, using current insulin dose, for a 6-day in-clinic IDD period, with blinded continuous glucose monitoring throughout the in-clinic periods.

***Results:*** We enrolled 21 participants (mean ± standard deviation age 57 ± 8 years; HbA1c 8.2% ± 0.9% [66 ± 9.8 mmol/M]) using U-100 insulin lispro (*n* = 11) or who switched to U-100 RHI (*n* = 10). Glycemic measures improved from the MDI to IDD period, including fasting blood glucose (BG), 141.2 ± 38.3 mg/dL (7.8 ± 2.1 mmol/L) versus 121.2 ± 35.0 mg/dL (6.7 ± 1.9 mmol/L; *P* = 0.002), respectively; 24-h mean BG, 137.0 ± 20.5 mg/dL (7.6 ± 1.1 mmol/L) versus 125.0 ± 16.5 mg/dL (6.9 ± 0.9 mmol/L; *P* = 0.004); and time in range (at 70–180 mg/dL; 3.9–10 mmol/L), 81.0% ± 14.4% versus 87.5% ± 10.6% (*P* = 0.008). No significant differences between MDIs and IDD use were recorded for time <70 mg/dL (1.6% ± 2.7% vs. 3.1% ± 2.7%, *P* = 0.08), CV%, or mean of daily differences. Mean amplitude of glycemic excursions was significantly lower with the IDD (*P* = 0.011). There were no significant differences between insulin lispro and RHI for any glycemic measure. No serious adverse events were recorded.

***Conclusions:*** In the context of this exploratory study, the IDD was safe and effective to administer insulin lispro and RHI for adults with T2D.

## Introduction

People with type 2 diabetes (T2D) may progress to requiring both basal and bolus (prandial) insulin therapy to attain glycemic control, thus necessitating multiple daily insulin injections (MDIs).^1,2^ However, glycated hemoglobin (HbA1c) targets are often not reached with MDI therapy.^3–5^ Moreover, results of surveys and other real-world studies indicate that nonadherence to insulin therapy is common, especially with more prescribed injections and as regimens become more complex.^6–9^ Indeed, taking more daily injections is an independent risk factor for nonadherence.^6^

An alternative to MDI therapy is the use of a wearable insulin pump delivering a continuous subcutaneous insulin infusion (CSII), long used for selected patients with type 1 diabetes and now more frequently being utilized by patients with T2D. The results of recent prospective randomized and retrospective studies indicate improved HbA1c and glycemic control with CSII therapy (either durable pump or patch pump), and often reduced insulin doses, when compared with MDI therapy.^10–16^

Rapid-acting insulin products such as insulin lispro, aspart, and glulisine are typically employed in insulin pumps. Since the time–action profile of rapid-acting insulin better mimics endogenous insulin compared with regular human insulin (RHI), it has been assumed that rapid-acting insulin should be used in pumps for treating patients with diabetes. However, these insulin analogs are typically more expensive than older prandial insulins such as RHI.

In the United States, many people struggle financially to support their insulin needs.^17–19^ RHI is much less expensive than many rapid-acting insulin analogs,^17,18^ and trial data suggest that patients with T2D may be appropriately controlled on human insulin.^17,20^ Nonetheless, while rapid-acting insulin products are approved for use in insulin pumps,^21,22^ RHI is not approved or recommended for pump use according to manufacturers' drug labeling.^22,23^

**FIG. 1.**
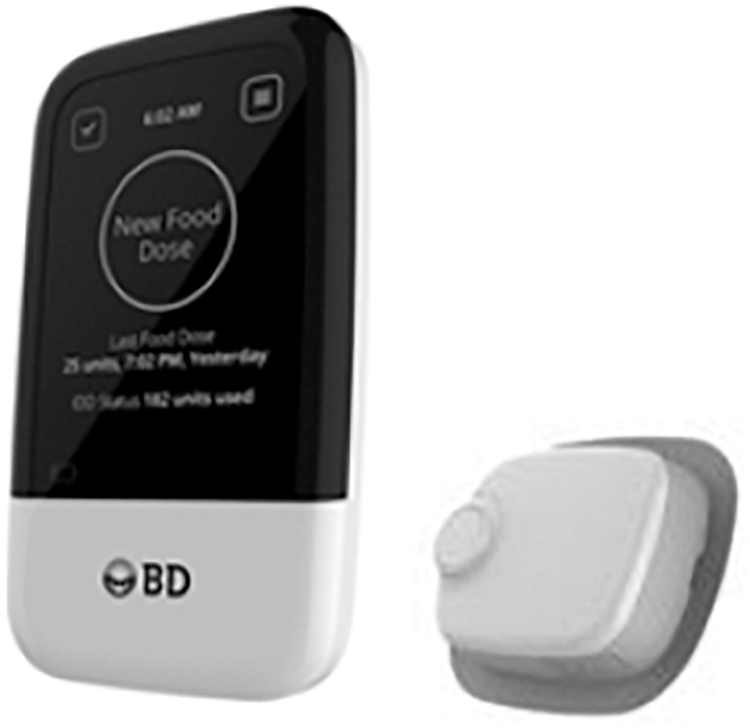
A novel insulin delivery device providing basal/bolus therapy with rapid-acting insulin or regular human insulin used in this study.

A novel insulin delivery device (IDD) has been developed to deliver rapid-acting insulin over ∼3 days as a continuous infusion (basal dose) in addition to bolus doses on demand for adults with T2D (Fig.1). The IDD's ability to deliver short-acting human insulin was also evaluated in this study. The aim of this exploratory pilot study was to assess the IDD performance, safety, effectiveness, and usability for providing rapid-acting or RHI over a total of 6 days in a clinic setting for adults with T2D.

## Methods

### Study design

This prospective, single-center open-label study included two in-clinic periods: (1) a 2- to 3-day in-clinic stay, during which participants received MDI therapy (MDI period), followed within 1 week by (2) a 6-day in-clinic stay, during which they received insulin through the IDD (IDD period). During the in-clinic periods, activity was limited to indoor options and meals were managed to match participants' usual intake, although a standardized breakfast meal was provided. Meal consistency was maintained using a standardized daily breakfast (carbohydrates: 50%–60%, proteins: 15%–18%, and fat: 18%–33%) and managed food intake for lunch and supper (similar in composition to their normal daily intake).

The IDD comprises two components: an investigational patch pump (oval, dimensions 43.5 × 53 × 14 mm, weight 25 g) and a wireless controller. The patch pump is worn on the body and is intended for continuous subcutaneous delivery of insulin over a 72-h period, using a user-adjusted basal delivery rate range of 10–70 U of insulin per day. The devices in this study provided user-initiated bolus dosing of 1–50 U per dose, configurable by either the wireless controller or the IDD side bolus buttons. The wireless controller is a separate electronic device used to communicate to and from the IDD. The insulin reservoir holds up to 300 U, and the IDD has an intended wear period of ∼3 days, up to a maximum of 84 h, and was replaced at least once during the 6-day IDD period.

The day before the start of each in-clinic period, participants visited the clinic and were supplied with a blinded continuous glucose monitor (CGM; Dexcom G4^®^ Platinum Professional; Dexcom, San Diego, CA), which they wore at home for ∼24 h and then throughout the subsequent in-clinic stay. They also used a blood glucose meter (BGM; CONTOUR^®^NEXT Blood Glucose Monitoring System; Ascensia Diabetes Care, Parsippany, NJ) at least four times daily, with regular monitoring by site staff and the study clinician.

### Participants

Adults (22–70 years old) with T2D were eligible for the study if they were using insulin therapy by MDIs for ≥6 months with recorded HbA1c of 7%–11% (53–97 mmol/M) within 30 days of enrollment. The MDI regimen had to be stable for ≥2 months and include at least three injections per day (≥1 basal insulin and ≥2 bolus/prandial insulin doses) with a mean maximum total daily dose of 90 U. Adults who were either taking insulin lispro (Humalog, 100 U/mL) or RHI (Humulin R, 100 U/mL) were recruited. For participants transitioning to RHI, a 7-day transition period was used. Participants could receive concomitant oral antidiabetic medications and noninsulin injectable antidiabetic agents, which were kept stable throughout. Other eligibility criteria included current use of BGM ≥2 times per day, willingness to have a CGM device applied during both at-home and in-clinic periods, and the ability to use BGM results to calibrate the CGM.

Key exclusion criteria were current use of premixed insulin, body–mass index (BMI) of <25 or ≥45 kg/m^2^, uncontrolled hypertension, or systemic corticosteroid use within 3 months of the study. We excluded women who were pregnant or breastfeeding and adults who would be poor candidates for a wearable IDD, such as individuals with poor eyesight and hand dexterity or who did not use technology or devices.

All participants provided written informed consent before the study eligibility assessments, and the protocol was approved by a research ethics board.

### Procedures and end points

During the IDD period, participants first received an IDD training session conducted by the pump trainer and then underwent an IDD proficiency test comprising a checklist of steps for the assembly, filling, priming, and operation of the IDD. Participants needed to successfully pass each step to remain in the study. The location of the first device placement was then randomly assigned to the left or right abdomen, and subsequent device placements alternated between left and right sides. Insulin doses as well as patients' individual dosing patterns and their timing were continued unchanged in the IDD period. Investigator discretion was allowed for adjustment to avoid hypoglycemia and hyperglycemia.

The study objectives were exploratory and included assessment of IDD insulin dose delivery, glycemic measures, and participant preferences for MDI versus IDD therapy, as well as comparison of glycemic profiles using either a rapid-acting insulin or RHI during MDI and IDD periods.

Several parameters of IDD usability and wearability were collected by study staff, including the IDD insertion success or failure, duration of wear (from time of application to time of removal), erythema or edema (before and after removal of the IDD), reasons for removal, bleeding upon removal, and integrity of the adhesive patch to the skin (daily, including just before IDD removal).

Study participants assessed the IDD wear comfort daily using a 5-item Likert scale and assessed pain on insertion, wear, and removal using a 0- to 10-point scale. Upon removal of the IDD, their preference for MDI versus IDD therapy was captured on a 150-mm visual analog scale (VAS) ranging from −75 mm (MDIs greatly preferred) to +75 mm (IDD greatly preferred).

### Statistical analyses

Since the study was exploratory, no formal sample size or statistical power calculation was performed. We summarized demographic and clinical characteristics of participants as well as glycemic and other outcomes using descriptive analyses, including frequencies and percentages for categorical variables and mean ± standard deviation (SD) or median (range) for variables measured on the continuous or interval scale.

Statistical comparisons of CGM or BGM data between the MDI and IDD study periods were made using linear mixed effect models to evaluate the period effects on glycemic measures. In the models, the participant was a random effect, and study period, time point, and insulin type were fixed effects. Two-way and three-way interactions between fixed effects were investigated; and interactions were removed from the model as no significant interactions were identified (all *P* ≥ 0.05). Models were also used to compute estimated mean and corresponding two-sided 95% confidence intervals (CIs) for responses of interest by study period. Bootstrapping was used to compute two-sided 95% CIs for responses per day within each period because of the imbalance in the number of days per period.

A similar model was used to compare participant-reported questionnaire results between the two insulin cohorts.

Statistical significance was defined as *P* < 0.05, and adjustments for multiple comparisons were made when appropriate using Tukey's method. Analyses were carried out using the R language for statistical computing (version 3.2.0 or newer, https://www.r-project.org).

## Results

### Baseline characteristics

Of 27 adults screened, 21 met study eligibility criteria and were enrolled, of whom 10 participants were switched from their current rapid-acting insulin regimen to the RHI cohort for study participation. Results for all 21 participants were included in the analyses, including those for a participant who discontinued the study prematurely for a nonmedical emergency after 53 h of wearing the second IDD.

Mean age in both insulin cohorts was 57 years (overall mean age 57.2 ± 7.9 years), with overall median diabetes duration of 15 years (range 5–34 years) and median MDI duration of 6 years (range 1–18 years), similar in both cohorts (Table 1). Mean BMI was 34.4 ± 6.0 kg/m^2^. Metformin was used as an adjunct therapy by 14 participants and in addition to metformin, 4 used an incretin therapy, 2 used an SGLT2 inhibitor therapy, and 5 used both.

**Table 1.** Characteristics and Diabetes-Related History of Study Participants

Variable	Insulin lispro (*n* = 11)	RHI*^a^*(*n* = 10)	All (*N* = 21)
Male sex, *n* (%)	4 (36.4)	5 (50.0)	9 (42.9)
Age, years	57.2 ± 7.5	57.2 ± 8.7	57.2 ± 7.9
Race, *n* (%)			
White	7 (63.6)	10 (100)	17 (81.0)
Black or African American	2 (18.2)	0	2 (9.5)
Asian	1 (9.1)	0	1 (4.8)
Other	1 (9.1)	0	1 (4.8)
Weight, kg	96.4 ± 11.6	102.7 ± 18.0	99.4 ± 15.0
BMI, kg/m^2^	32.6 ± 5.8	36.4 ± 6.0	34.4 ± 6.0
HbA1c, %	8.2 ± 0.7	8.3 ± 1.1	8.2 ± 0.9
HbA1c, mmol/M	66 ± 7.7	67 ± 12.0	66 ± 9.8
Diabetes duration, median (IQR), years	16.0 (12.2–24.0)	14.5 (8.8–20.4)	15.0 (11.1–24.0)
Range, years	5–34	5–33	5–34
MDI duration, median (IQR), years	5.0 (3.8–8.8)	6.5 (3.9–13.3)	5.8 (3.8–10.9)
Range, years	1–18	3–15	1–18
Used insulin pen, *n* (%)	11 (100)	10 (100)	21 (100)
Basal injections/day (range)	1.3 ± 0.9 (1–4)	1.3 ± 0.7 (1–3)	1.3 ± 0.8 (1–4)
Bolus injections/day (range)	2.6 ± 0.5 (2–3)	2.6 ± 0.7 (1–3)	2.6 ± 0.6 (1–3)

Data are mean ± SD unless otherwise indicated.

^a^ One participant discontinued the study prematurely, after 53 h of wearing the second insulin delivery device, but was included in the results.

HbA1c, glycated hemoglobin; IQR, interquartile range; MDIs, multiple daily insulin injections; RHI, regular human insulin; SD, standard deviation.

In the MDI period, the mean total daily insulin dose was 57.6 ± 20.3 U (insulin lispro cohort 53.3 ± 21.5 U and regular insulin cohort 62.5 ± 18.0 U; Table 2). During the IDD period, mean total daily insulin doses did not change significantly in either cohort, but the mean basal insulin dose did increase significantly and the mean bolus insulin dose did decrease significantly overall (Table 2).

**Table 2.** Mean (± Standard Deviation) Daily Insulin Doses During the Two Study Periods

	2- to 3-Day MDI period		6-Day IDD period		All participants by period	
	Insulin lispro (*n* = 11)	RHI (*n* = 10)	Insulin lispro (*n* = 11)	RHI (*n* = 10)	MDI period (*n* = 21)	IDD period (*n* = 21)
Basal dose, units	27.5 ± 11.2	35.5 ± 15.6	33.5 ± 12.2	39.5 ± 18.5	31.2 ± 13.9	36.4 ± 15.8^*^
Bolus dose, units	25.8 ± 14.4	27.0 ± 15.2	22.0 ± 14.0	25.1 ± 13.7	26.4 ± 14.7	23.5 ± 13.9^*^
Total dose, units	53.3 ± 21.5	62.5 ± 18.0	55.5 ± 22.6	64.6 ± 19.0	57.6 ± 20.3	59.9 ± 21.4

^*^ *P* < 0.05 for comparison between MDI and IDD periods for all participants.

IDD, insulin delivery device.

### Glycemic measures

During the IDD period, the mean values of daily fasting blood glucose (BG) and 24-h BG were significantly lower (*P* ≤ 0.004) compared with the MDI period (Table 3). Similarly, the mean percentage of CGM readings in range defined as 70–180 mg/dL (3.9–10 mmol/L) was significantly greater (*P* = 0.008), and the mean percentage of BG values in hyperglycemia >180 mg/dL (>10 mmol/L) was significantly lower during the IDD period (*P* = 0.002). The percentages of BG values in hypoglycemia did not differ significantly between study periods (Table 3).

**Table 3.** Glycemic Measures and Glycemic Variability During 2- to 3-Day Multiple Daily Insulin Injection and 6-Day Insulin Delivery Device in-Clinic Periods

BG variable	MDI period by insulin type		IDD period by insulin type		All participants by period (*n* = 21)		
	Insulin lispro (*n* = 11)	RHI (*n* = 10)	Insulin lispro (*n* = 11)	RHI (*n* = 10)	MDI period (*n* = 21)	IDD period (*n* = 21)	P value MDI vs. IDD
Fasting BG, mg/dL (mmol/L)^a^	144.6 ± 47.2 (8.0 ± 2.6)	137.5 ± 27.4 (7.6 ± 1.5)	121.6 ± 45.3 (6.8 ± 2.5)	120.7 ± 21.0 (6.7 ± 1.2)	141.2 ± 38.3 (7.8 ± 2.1)	121.2 ± 35.0 (6.7 ± 1.9)	0.002
24-H BG, mg/dL (mmol/L)	143.8 ± 23.5 (8.0 ± 1.3)	129.4 ± 14.0 (7.2 ± 0.8)	126.1 ± 19.9 (7.0 ± 1.1)	123.8 ± 12.8 (6.9 ± 0.7)	137.0 ± 20.5 (7.6 ± 1.1)	125.0 ± 16.5 (6.9 ± 0.9)	0.004
BG values in range,^b^ %	77.6 ± 18.5	84.7 ± 7.3	87.5 ± 12.3	87.5 ± 9.0	81.0 ± 14.4	87.5 ± 10.6	0.008
BG values >180 mg/dL (>10 mmol/L), %	21.7 ± 17.9	12.7 ± 7.2	9.8 ± 11.9	9.0 ± 7.6	17.4 ± 14.3	9.4 ± 9.9	0.002
BG values <70 mg/dL (<3.9 mmol/L), %	0.7 ± 0.9	2.6 ± 3.6	2.7 ± 2.6	3.5 ± 3.0	1.6 ± 2.7	3.1 ± 2.8	0.084
BG values <50 mg/dL (<2.8 mmol/L), %	0.03 ± 0.09	0.15 ± 0.35	0.04 ± 0.09	0.14 ± 0.24	0.09 ± 0.25	0.09 ± 0.18	0.99
Coefficient of variation (SD/mean)	0.27 ± 0.06	0.30 ± 0.06	0.28 ± 0.06	0.29 ± 0.05	0.29 ± 0.06	0.28 ± 0.06	0.87
MODD, mg/dL (mmol/L)	29.3 ± 11.0 (1.6 ± 0.6)	35.8 ± 10.5 (2.0 ± 0.6)	31.0 ± 12.8 (1.7 ± 0.7)	31.0 ± 13.0 (1.7 ± 0.7)	32.4 ± 11.0 (1.8 ± 0.6)	31.0 ± 12.6 (1.7 ± 0.7)	0.59
MAGE, mg/dL (mmol/L)	105.4 ± 23.8 (5.9 ± 1.3)	101.8 ± 20.4 (5.7 ± 1.1)	92.4 ± 12.6 (5.1 ± 0.7)	92.9 ± 9.7 (5.2 ± 0.5)	103.7 ± 11.1 (5.8 ± 1.6)	92.6 ± 11.0 (5.1 ± 0.6)	0.011

Reported values are mean ± SD. *P* value is for comparison between MDI and IDD periods for all participants.

^a^ Fasting BG data were taken using the blood glucose meter from a single fingerstick value before breakfast. All other glycemic measures were recorded using the continuous glucose monitor.

^b^ BG values in range defined as 70–180 mg/dL (3.9–10 mmol/L).

BG, blood glucose; MAGE, mean amplitude of glycemic excursion; MODD, mean of daily differences.

Of the measures of glycemic variability, the mean amplitude of glycemic excursion (MAGE) results was significantly lower (*P* = 0.011) during the IDD period, although the coefficient of variation and the mean of daily differences were not significantly different between MDI and IDD periods (Table 3).

There were no significant differences between the two insulin cohorts in any of the glycemic measures during either the MDI or IDD in-clinic period.

### Device assessments

Twenty of the 21 participants passed on the first IDD proficiency test, while 1 of the 21 participants failed the initial attempt (the steps required to fill the IDD with diluent) but passed on the second attempt. The IDD wear duration is summarized in Table 4. IDD-related adverse effects such as erythema, edema, and bleeding were uncommon and rated as mild (Table 4). There were no differences in device assessment outcomes between the two insulin cohorts. There were no cases of severe hyperglycemia or infection.

**Table 4.** Wear Duration and Incidence of Insulin Delivery Device-Related Erythema, Edema, and Bleeding for 48 Devices Applied During the Study

Variable	Insulin lispro (*n* = 25 IDD)	RHI (*n* = 23 IDD)	All (*n* = 48 IDD)
Device wear duration, hours			
Median (IQR)	71.7 (55.6–73.1)	72.3 (52.9–72.7)	71.7 (55.1–72.9)
Range	28.7–74.1	10.5–74.5	10.5–74.5
Erythema before IDD insertion^a^			
0—none	22 (88.0)	23 (100)	45 (93.8)
1—very slight (barely perceptible)	3 (12.0)	0	3 (6.2)
Erythema after IDD removal^a^			
0—none	19 (76.0)	16 (69.6)	35 (72.9)
1—very slight (barely perceptible)	6 (24.0)	7 (30.4)	13 (27.1)
Edema before IDD insertion^a^			
0—none	24 (96.0)	23 (100)	47 (97.9)
1—very slight (barely perceptible)	1 (4.0)	0	1 (2.1)
Edema after IDD removal^a^			
0—none	24 (96.0)	22 (95.7)	46 (95.8)
1—very slight (barely perceptible)	1 (4.0)	1 (4.3)	2 (4.2)
Bleeding after IDD removal^a^			
0—none	25 (100)	22 (95.7)	47 (97.9)
1—just visible spot of red	0	0	0
2—a drop of red blood	0	0	0
3—a continuing ooze of red blood	0	1 (4.3)	1 (2.1)
4—significant bleeding from site	0	0	0

Reported values are *n* (%) unless otherwise noted.

^a^ The extent of erythema, edema, and bleeding, each scored from 0 (none) to 4 (worst).

During the study, 4 of 48 IDDs were replaced for malfunctions recognized by the user or study staff, including 1 wireless controller connection issue and 3 pump stalls with IDD status error. In addition, three kinked catheters were detected upon IDD removal, although it is unlikely that insulin delivery was impaired in these three cases.

### Participant-reported measures

At the end of the IDD period, all participants rated the IDD wear comfort as either comfortable or very comfortable (Table 5). Nearly all VAS pain scores upon IDD insertion (96%) were 0 of 10 (no pain), and pain during removal of the IDD was rated 0 of 10 in 92% of instances. The maximum pain score recorded was 2 of 10 (two instances during IDD removal); the other five pain scores >0 were rated as 1.

**Table 5.** Participant-Reported Measures: Wear Comfort, Pain Scores, and Preference for Multiple Daily Insulin Injections Versus Insulin Delivery Device^a^

Variable	Insulin lispro	RHI	All
Wear comfort after IDD application			
Very comfortable (score of 1)	19 (76.0)	17 (73.9)	36 (75.0)
Comfortable (score of 2)	6 (24.0)	6 (26.1)	12 (25.0)
Wear comfort, day 0			
Very comfortable (score of 1)	16 (80.0)	18 (85.7)	34 (82.9)
Comfortable (score of 2)	4 (20.0)	3 (14.3)	7 (17.1)
Wear comfort, day 1			
Very comfortable (score of 1)	18 (81.8)	17 (81.0)	35 (81.4)
Comfortable (score of 2)	4 (18.2)	4 (19.0)	8 (18.6)
Wear comfort, day 2			
Very comfortable (score of 1)	19 (86.4)	16 (88.9)	35 (87.5)
Comfortable (score of 2)	3 (13.6)	2 (11.1)	5 (12.5)
Wear comfort, day 3			
Very comfortable (score of 1)	9 (81.8)	6 (75.0)	15 (78.9)
Comfortable (score of 2)	2 (18.2)	2 (25.0)	4 (21.1)
Wear comfort before IDD removal			
Very comfortable (score of 1)	23 (92.0)	19 (82.6)	42 (87.5)
Comfortable (score of 2)	2 (8.0)	4 (17.4)	6 (12.5)
Pain score before IDD application			
Pain score of 0	25 (100)	23 (100)	48 (100)
Pain score after IDD application			
Pain score of 0	24 (96.0)	22 (95.7)	46 (95.8)
Pain score of 1	1 (4.0)	1 (4.3)	2 (4.2)
Pain score during wear days			
Pain score of 0	74 (98.7)	68 (100)	142 (99.3)
Pain score of 1	1 (1.3)	0	1 (0.7)
Pain score before IDD removal			
Pain score of 0	25 (100)	23 (100)	48 (100)
Pain score during IDD removal			
Pain score of 0	24 (96.0)	20 (87.0)	44 (91.7)
Pain score of 1	1 (4.0)	1 (4.3)	2 (4.2)
Pain score of 2	0	2 (8.7)	2 (4.2)
Relative VAS for preference			
Median (IQR)	+75 (+38 to 75)	+75 (+58 to 75)	+75 (+49 to 75)
Range	+23 to 75	+15 to 75	+15 to 75
Mean (SD)	+59 (22)	+64 (19)	+61 (20)

Reported values are *n* (%) unless otherwise noted.

^a^ IDD wear comfort was scored from 1 (very comfortable) to 5 (very uncomfortable); pain was scored from 0 (none) to 10 (very painful); and preference was scored on a 150-mm VAS ranging from −75 mm (MDIs greatly preferred) to +75 mm (IDD greatly preferred)

VAS, visual analog scale.

All participants reported preference for the IDD over MDI therapy, with a mean score of +61 mm (SD ±20) and median score of +75 mm (interquartile range +49 to 75) of a possible +75 mm (score of 0 representing no difference; Table 5).

There were no significant differences between the two insulin cohorts for these participant-reported outcomes.

## Discussion

In the context of this small pilot study, the use of the investigational IDD appeared to be safe and effective, delivering insulin as intended for adults with T2D. During the in-clinic IDD period, fasting BG, 24-h mean BG, and time in glycemic range were all significantly improved compared with values observed during the prior in-clinic period of MDI therapy, with no change in total daily insulin dose. Furthermore, while using the IDD, participants experienced significantly less time in the hyperglycemic range, with no difference in time spent in hypoglycemia, compared with the MDI period. The MAGE results were also improved during the IDD period. Importantly, we found no significant differences between the two insulin cohorts for any glycemic measure or participant-reported outcome. Preference for the wearable IDD was rated highly by participants compared with their usual MDI therapy.

The use of CGM enabled us to assess 24-h glycemic profiles during both MDI and IDD in-clinic periods.^24^ Prior studies have used CGM to evaluate glycemic measures with insulin pump therapy for T2D; however, these studies have not assessed the use of RHI.^25–28^ Lane et al.^29^ used periodic 72-h CGM during their 1-year study of 20 patients with severe insulin-resistant T2D who were administered U500 insulin by CSII. They found that the percentage of time spent in the glycemic range improved significantly, and there was no increase in hypoglycemia, compared with prior MDI regimens.

In the present study, both insulin cohorts showed similar outcomes for multiple glycemic measures. To the best of our knowledge, this report is the first to use CGM data in patients with T2D to study and compare the effects of RHI with those of a rapid-acting insulin analog, whether administered by MDIs or by a novel IDD. Other strengths of the study include the comparison of glycemic measures using MDIs versus IDD under similar in-clinic settings and the careful monitoring conducted during the in-clinic periods. The fact that there was no change in total daily insulin dose was expected considering the short (6-day) IDD period. The increase seen in the proportion of basal to bolus dose has been described in prior studies of pump therapy in patients with T2D^11^ and may be related to either more consistent action of CSII-delivered basal insulin or improved adherence to prescribed bolus dosing.

The frequency of device malfunction has led to additional efforts to mitigate the chance of future wireless connection issues or pump stall errors such as those occurring during the study. Updates to the design and software of the IDD have been implemented since study completion. The three kinked catheters identified upon IDD removal did not appear to have resulted in substantial (if any) disruption of insulin flow based on the associated glycemic responses.

Several study limitations should be considered when evaluating our findings. This study was exploratory and not powered for any one specific outcome measure. The sample size was relatively small (*N* = 21) and included largely white participants with a relatively high time in the glycemic range at baseline, limiting the generalizability of the findings. We did not use a crossover design, and subgroup comparisons between insulin lispro and RHI cohorts (*n* = 11 vs. *n* = 10) may be inappropriate for statistical analyses. Finally, the outcomes were assessed under in-clinic supervision, with controlled food intake and somewhat limited physical activity, while outcomes in the real-world setting may be different.

In conclusion, because of the nature of the study design (i.e., noncrossover in-clinic study) and reasons cited above, caution should be used when interpreting our findings, and the results should not be overgeneralized. We acknowledge that these were comparisons of small subgroups. Nonetheless, these data suggest that RHI is safe to use in the IDD and could potentially be used by adults to manage their T2D. In the context of this exploratory pilot study, the IDD, used with either insulin lispro or RHI in adult patients with T2D, was safe and effective in the short term. Larger studies are warranted to further investigate these findings.
